# Public health round-up

**DOI:** 10.2471/BLT.22.010722

**Published:** 2022-07-01

**Health care under attack in Ukraine**

World Health Organization (WHO) Unit Head of Emergency Operations Centre and Incident Manager for Ukraine, Ian Clarke, and WHO Executive Director for Health Emergencies, Mike Ryan, assess the damage done to a building at the Makariv District Hospital in Ukraine. As of 2 June, there had been 269 verified attacks on health care infrastructure, material and staff, resulting in at least 76 deaths of health workers and the injury of 59.

**Figure:**
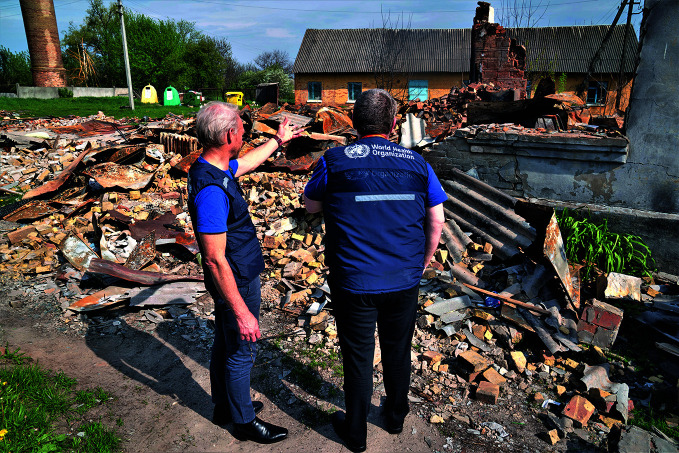
WHO/Andrei Krepkih

## Paediatric hepatitis of unknown origin

An outbreak of severe acute hepatitis of unknown origin in children was reported across five WHO regions. Initial reports in April from the United Kingdom of Great Britain and Northern Ireland were followed by reports of cases worldwide. Some 650 probable cases of acute hepatitis in children were reported to WHO from 33 Member States between 5 April and 26 May 2022.

As of mid-June, the origin of the disease had still not been established. None of the viruses that commonly cause acute viral hepatitis had been detected in the patients. Also of concern was the clinical severity of the cases and the proportion of children developing acute liver failure. As of 27 May, at least 38 of the children in the 650 cases reported to WHO had required liver transplants, and nine of the 650 had died.

Efforts are focusing on determining the cause of the illness to be able to further refine control and prevention actions. A possible association with circulating adenoviruses is being investigated, including possible increased susceptibility to such viruses among young children following a lower level of circulation of adenovirus during the coronavirus disease 2019 (COVID-19) pandemic. The potential emergence of a novel adenovirus is also being considered, among other possibilities. Hypotheses related to side effects from COVID-19 vaccines are currently not supported as most of the affected children did not receive these vaccines.

Clinical and public health incident responses have been activated across the affected regions to coordinate case finding with investigation into the cause of illness and WHO is continuing to support information sharing with professional networks and specialist liver units. WHO is also developing guidance to support Member States with diagnostics, case investigation and reporting, clinical characterization and clinical management of acute liver failure in children.

https://bit.ly/3Hg0D2A

## Monkeypox outbreak

The abrupt appearance of monkeypox in several non-endemic countries raised concerns. As of 8 June, 1285 laboratory confirmed cases and one probable case had been reported in 28 Member States across four WHO regions where monkeypox is not usual or had not previously been reported. Only one person had travelled to a country where monkeypox is endemic.

The geographically dispersed nature of the cases suggests that the virus may have been spreading undetected in communities for some time.

On 3 June the WHO R&D Blueprint initiative convened a meeting of experts to discuss knowledge gaps and research priorities. Recommendations emerging from the two-day consultation included the need for improved control of monkeypox in endemic countries and strengthened collaboration between researchers in endemic and non-endemic countries.

WHO published interim guidance for the clinical management and infection prevention and control of the disease on 10 June and interim vaccine and immunization guidance on 14 June.

https://bit.ly/3NNg8BL

## Haemorrhagic fever outbreak in Iraq

Iraq saw a significant increase in cases of Crimean-Congo haemorrhagic fever (CCHF) in the first five months of 2022. A further increase can be expected in July during the religious holiday of Eid al-Adha.

According to a 1 June report, Iraqi health authorities notified WHO of 212 people being infected. Ninety-seven of those cases were laboratory confirmed, up from 33 laboratory-confirmed cases reported in the first five months of 2021. Of the 212 people infected in 2022, 27 died. Most of the cases were reported in the south-east of the country with nearly 50% in Dhi Qar Governorate, although there were also cases in the north-west.

There is a perceived risk of further spread due to population movement and the slaughtering of camels, cows and sheep during the Eid al-Adha holiday (CCHF is caused by a tick-borne *Nairovirus* that is primarily transmitted to people through contact with livestock).

Additionally, international cross-border transmission is a possibility given the increased population movement and possible animal exportation associated with the holiday.

CCHF is characterized by severe viral haemorrhagic fever and has a case fatality rate ranging from 10% to 40%.

https://bit.ly/3Qktebq

## Novel pathogens report

The first preliminary report from the Scientific Advisory Group for the Origins of Novel Pathogens to WHO was released on 9 June. The report provides initial recommendations for the development of a global framework to study emerging and re-emerging pathogens with pandemic potential as well as preliminary recommendations regarding urgent studies needed to better understand the origins of the COVID-19 pandemic.

The group noted that data required to establish a complete understanding of how the COVID-19 pandemic began have yet to be made available. This includes data required to evaluate the possible role played by a laboratory acting as “a pathway” for severe acute respiratory syndrome coronavirus 2 (SARS-CoV-2) into the human population. The report recommends further investigations into this and all other possible pathways.

The group underlines the fact that it was not formed to find the origins of SARS-CoV-2 but rather to advise on studies that are necessary to gather evidence to better understand the origins of SARS-CoV-2, and more broadly, the origins of emerging and re-emerging future infectious diseases.

https://bit.ly/39fbD3X

## WHO Ukraine response

The war in Ukraine entered its fourth month, with the country struggling to meet the increased need for health care particularly in areas of active conflict. As of 2 June, there had been 269 verified attacks on health care, resulting in the death of 76 health workers. Some health facilities have been destroyed, while others have been overwhelmed by people seeking care for trauma and injuries resulting directly from the war.

WHO has responded by establishing hubs in areas close to the conflict to rapidly reach the areas of greatest need, by increasing the number of staff committed, and by repurposing systems including WHO logistics.

This has enabled delivery of over 543 metric tonnes of medical supplies and equipment to the country which are being distributed mostly in the east, south and northern oblasts. Supplies provided included trauma surgery supplies, ambulances, Ukrainian-made ventilators able to function even when power fails, electric generators and oxygen equipment, including the oxygen plants hospitals need to function autonomously.

To support this and other interventions, WHO launched an updated appeal for US$ 147.5 million to respond to Ukraine’s increasing humanitarian needs, ensure immediate health-care delivery and support longer term health system resilience.

https://bit.ly/3y1BBl7

## Promoting cancer care innovation

WHO joined forces with the American Society of Clinical Oncology (ASCO), an association of physicians and oncology professionals, to develop and promote cancer care innovations specifically designed to take account of the resource constraints encountered in many lower-income countries. The aim is to overcome some of the current inequities that exist in access to cancer care.

Announced 4 June, the new partnership will enable ASCO and WHO to develop a coordinated approach to supporting WHO Member States in their efforts to improve access to quality care, notably by linking facility-level quality improvement activities with national strategies.

Over the last two decades disparities in access to care have increased, with high-income countries seeing markedly improved survival rates due to innovation and reliable access to high-quality care that is largely unavailable in low-income countries and marginalized communities.

https://bit.ly/3HfNXsC

**Cover photo**

A young man carrying water in the city of Toretsk, Donetsk, Ukraine.

**Figure:**
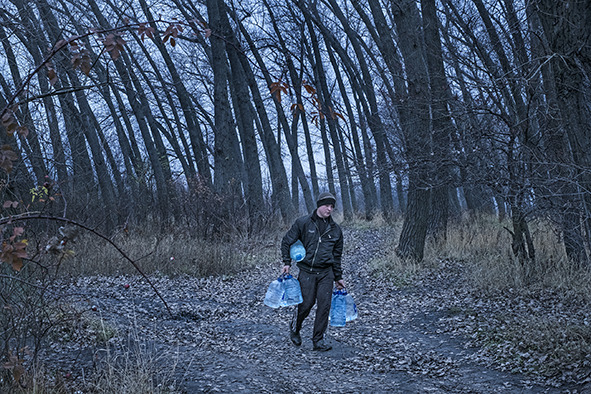
UNICEF/Gilbertson

## New skin-related neglected tropical diseases framework

WHO published a strategic framework for skin-related neglected tropical diseases (skin NTDs) that identifies opportunities to integrate approaches for control and management, including common learning platforms, capacity-building for case detection and delivery of treatment.

Skin NTDs, ranging from Buruli ulcer to yaws, afflict hundreds of millions of people, and cause immense discomfort, suffering, stigmatization and mental distress.

https://bit.ly/3tAyRbs

## The multiple benefits of walking and cycling

A new WHO report on walking and cycling shows how investment in policies promoting these activities can play a crucial role in improving health and the environment.

Launched on 7 June, the report provides a set of recommendations to reallocate space for cycling and walking, improve active mobility infrastructure, increase cyclist and pedestrian safety to reduce fatalities, develop national cycling policies, and integrate cycling into health policies and urban and transport planning.

https://bit.ly/3MMk6ZV

**Looking ahead**

4–5 July, Ukraine Recovery Conference. Lugano, Switzerland. https://bit.ly/3mseUzL

22 July, World Brain Day 2022; Brain Health for All. https://bit.ly/3xeXMT8

31 August–2 September, Health-enhancing physical activity Europe 2022 Conference.

https://bit.ly/3tEGL3H

